# Exploring midwives’ experiences of implementing the Labour Hopscotch Framework: A midwifery innovation

**DOI:** 10.18332/ejm/146081

**Published:** 2022-04-04

**Authors:** Denise O’Brien, Barbara Coughlan, Sinead Thompson, Lorraine Carroll, Lucille Sheehy, Mary Brosnan, Martina Cronin, Teresa McCreery, Jean Doherty

**Affiliations:** 1 School of Nursing, Midwifery and Health Systems, University College Dublin, Dublin, Ireland; 2 National Maternity Hospital, Dublin, Ireland

**Keywords:** physiological birth, active birth, midwife as coach

## Abstract

**INTRODUCTION:**

Midwives are ideally placed to promote physiological birth and improve women’s birth experiences. Freedom of movement in labor is highly recommended as it reduces a need for obstetric interventions in labor and prevents and corrects labor complications, such as poor progress and malposition of the fetus. The Labour Hopscotch Framework (LHF) provides women and midwives with a visual depiction of the steps they can undertake to remain active and, in this way, support physiological birth processes. The objective of this study was to explore midwives’ experiences of supporting women during labor with the Labour Hopscotch Framework and identify any improvements necessary to the Labour Hopscotch Framework.

**METHODS:**

A two phased mixed-method sequential explanatory design study consisting of a survey (women, n=809 and partners, n=759) and focus group (n=8 midwives) was completed to evaluate the LHF following its implementation. This article presents the findings reporting midwives’ perceptions of using the Labour Hopscotch Framework with women and their birthing partners. The setting was a large urban teaching maternity hospital in Dublin, Ireland, where eight midwives practiced in the following areas: labor suite, antenatal unit, and community midwifery.

**RESULTS:**

The Labour Hopscotch Framework was described as beneficial in promoting physiological birth, using a creative, attractive visual depiction to guide women in, and before, labor. The Labour Hopscotch Framework was deemed helpful in increasing midwifery students and newly qualified midwives’ confidence to provide women with tangible, supportive assistance during labor and increased partners’ involvement in the labor process.

**CONCLUSIONS:**

Labour Hopscotch Framework should be more widely promoted to all women attending the hospital for maternity care and a clear explanation of each step given and demonstrated to increase women’s understanding of the steps within. Labour Hopscotch training should be included in midwifery education programs.

## INTRODUCTION

Midwives are passionate about making a difference for women, empowering them through education and supporting them to realize their plans for birth^1^. To support women to achieve a positive childbirth experience, the World Health Organization recommends mobilizing and upright positions during labor, supportive birth partners, and a kind and competent caregiver^2^. Freedom of movement is of central importance to many birthing women^3-6^ and highly recommended^3,7^. Flexible sacrum birthing positions can reduce the duration of the first stage of labor^8^ and the second stage of labor^3,4^, as well as reducing interventions such as severe perineal trauma, operative vaginal birth, caesarean section, and episiotomy^1^. Midwifery support through encouragement of freedom of movement is an integral aspect of midwifery care during labor^8-10^, as a lack of knowledge among women regarding the use of non-supine positions, and its effect on physiological birth, has been noted by midwives in the Dutch study of Thompson et al.^4^. Alternative therapies and non-pharmacological methods of pain relief are an integral aspect of midwifery support for physiological birth^7,10^.

Only by working with midwifery knowledge/evidence base can midwives support physiological birth and achieve the recommendations of the WHO 2018^2^ guideline for positive birth. In 2018, the national rate of caesarean section in Ireland was 31.2% from a total of 61655 births^11^. In response to the increased numbers of interventions reported, particularly epidural rates which were 57%^12^ at the time, senior midwifery management in the research site encouraged midwifery practitioners to consider developing innovations that could reduce rates of interventions and facilitate normal physiological birth for women. Subsequently one of the community midwives designed and produced a visual framework entitled ‘Labour Hopscotch Framework’ (LHF) that is intended to inform and empower women and their birth partners about measures/steps that could facilitate a physiological birth.

Details are available about the LHF in all the antenatal care options (clinics) and midwives inform women and their birthing partners about the framework at the booking appointment. LHF is incorporated into antenatal education classes with the intention of enabling women to prepare mentally and physically for labor. This is important as it means women can practice with their birthing partners and learn about the use of the robozzo scarf, breathing techniques, lunges, and squats all of which are important for active birth. In addition, education and training sessions have been provided to midwifery and other members of the multidisciplinary team about the LHF. Following the implementation of the LHF in 2016, a decision was made to conduct an output evaluation of the Labour hopscotch framework in 2017 and the final report was published in 2019^13^.

The conceptual thinking, research findings and clinical skills associated with the LHF^13^ are embedded into the undergraduate and post graduate midwifery curriculum in The School of Nursing Midwifery and Health systems, University College Dublin. Following a presentation of the project research findings^12^, the Department of Health, Ireland, through The National Women’s Infant Program (NWIP) supported a national implementation of the LHF across all maternity units in Ireland. Subsequently during 2020–2021, educational training and workshops were offered and LHF is available in 10 of 19 maternity units. The national roll out of the LHF was delayed and impacted by the current COVID-19 pandemic so virtual education occurred when necessary. The Health Service Executive Ireland included the LHF into The National Standards for Antenatal Education in Ireland (2020)^14^.

## METHODS

### Study design

**Figure 1:**
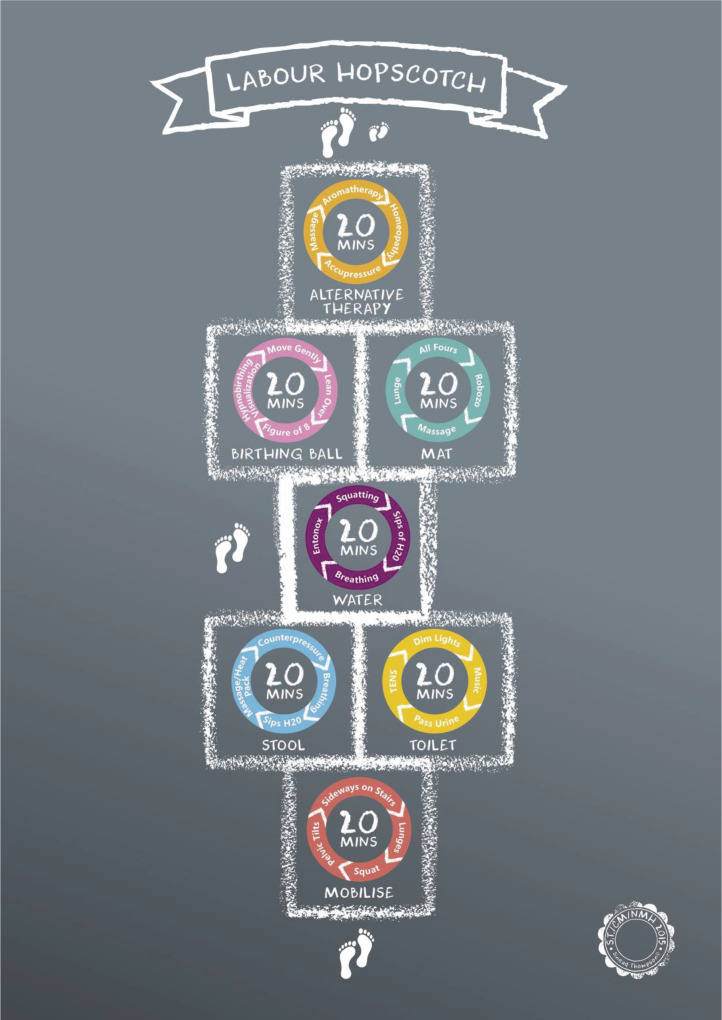
Labour Hopscotch Framework

The framework (Figure 1) was designed by a community midwife in the research site developed from both an understanding of the physiology of labor and midwifery knowledge and expertise gained from many years of supporting women during childbirth. The fundamental principle of the LHF is to inform women, their partners and midwives of the importance of the steps necessary to remain active during pregnancy and labor and in this way possibly reduce the rate of epidurals. An appropriate time-frame is provided for each step and is illustrated in a sequential manner that is matched with the progression of labor as demonstrated in Figure 1. The steps include the use of mobilization, positioning, water-therapy and non-pharmacological methods of pain relief. Women start at the bottom of the hopscotch as they are more active and mobile. As labor progresses, they advance towards the baby’s footprints. The framework recommends 20 minutes for each step, and flexibility is displayed within the steps, considering equipment availability and maternal comfort levels. The framework is designed to ensure the steps can be used antenatally, as part of training for active birth, and during labor.

**Figure 2:**
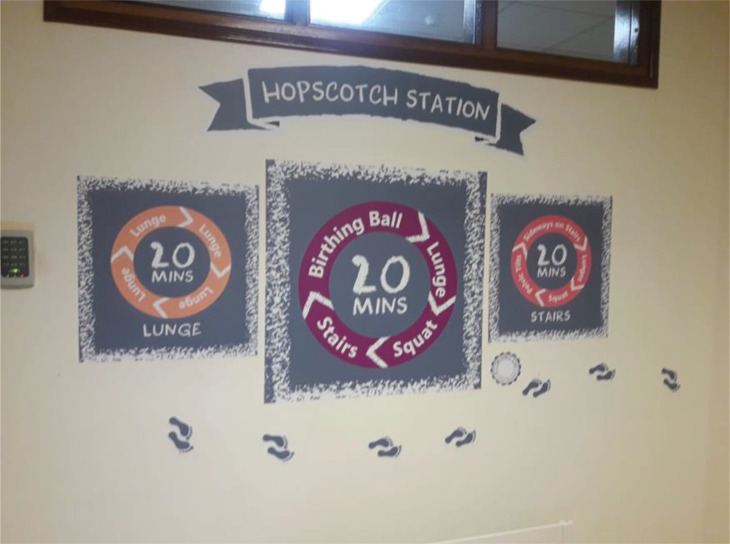
Labour Hopscotch station on the antenatal unit

To maximize the beneficial effects of the Labour Hopscotch, women need to be fully informed, therefore, to support shared decision-making, detail is readily available online on the hospital webpage for women to download. Visual images of the framework are displayed in each area of the hospital (Figure 2).

The aim of the study is to explore midwives’ experiences of supporting women during labor with the LHF and identify any improvements necessary before a national implementation plan was commenced.

### Setting

This study took place in a busy urban teaching maternity hospital which has an annual birth rate of approximately eight thousand births (8434 in 2018; 9400 in 2017). The hospital offers obstetric-led, midwifery-led and community/domino midwifery care (which includes the option for homebirth). The normal vaginal delivery rate of 57% is higher than the national average of 53.4% and the caesarean section rate of 28.7% is significantly lower than the national rate of 31.2%^11^. At the time of the study, the research site’s epidural rate was 57% in 2017 and 52% in 2018^12^.

### Data collection

The study was conducted over 18 months; the mixed-methods sequential approach adopted consisted of a survey and a focus group for data collection. This article presents findings from the focus group meeting with midwives and student midwives. Participants who expressed an interest in taking part in the study completed written consent and signed a confidentiality agreement before the meeting. The meeting was held at time that suited practicing midwives, in total 7 midwives and 1 student of midwifery participated in the focus group meeting. A topic guide was devised by DOB based on the findings from the survey results. The meeting was transcribed verbatim by one of the research team (JD) and participants were provided with a copy for their records. The transcript was reviewed by the team prior to data analysis. To ensure confidentiality, pseudonyms are adopted throughout the presentation of the findings.

### Data analysis

Data from the focus group transcript were analyzed using qualitative content analysis by DOB. An inductive, data-driven content analysis approach was adopted^15^. A member of the research team transcribed the transcript to maximize the level of engagement with the data. The transcript was read several times before the data were coded, and notes were made on the original transcripts of the identified themes and similarities. Prior to coding, the audio recording of the meeting was also listened to several times. A coding frame was generated in a manner that was both concept- and data-driven. The approach recommended by Mayring^16^ was adhered to when generating the coding frame. The software package NVIVO 9 was used to support the data analysis process. The codes and categories were refined and finalized after discussion between research team members.

## RESULTS

The demographic characteristics of the midwives and student of midwifery who participated in the focus group are presented in Table 1. Eight themes emerged from the data, three of which related to requested changes and are not reported here as they have since been implemented. The five themes presented here are: perceived value of the Labour Hopscotch Framework; implementation and use of the Labour Hopscotch Framework; relationships, barriers and challenges to using the Labour Hopscotch; education in relation to the Labour Hopscotch Framework.

**Table 1:** Demographics characteristics of focus group participants

*Variable*	*Category*	*n (%)*
**Profession**	Staff midwife	7 (87.5)
	Student midwife	1 (12.5)
**Area of practice**	Community midwife	1 (12.5)
	Delivery ward	6 (75.0)
	Antenatal ward	1 (12.5)
**Age** (years)	20–29	5 (62.5)
	30–39	2 (25.0)
	40–49	0 (0)
	50–59	1 (12.5)
**Years of experience**	Minimum	0
	Maximum	35
**Trained in Ireland**	Yes	7 (87.5)
	No	1 (12.5)
**Trained in research site**	Yes	5 (62.5)
	No	3 (37.5)

### Perceived value of the Labour Hopscotch Framework

Participating midwives described the Labour Hopscotch framework in terms that highlighted the activities they associated with it, such as ‘a circuit in a gym’. Indeed, midwives considered the core concept behind the LHF was women remaining ‘moving’ and ‘staying active’:

*‘I don't think it matters what type of lunge that they [women] do, like if they're lunging, they're moving, that's what matters, for me that's the idea behind it.’* (Kim)

*‘If you ever do a circuit in a gym or a circuit ... so that idea that this is what we do, we're always moving, and so you have a choice to do this or this or this.’* (Brenda)

Participating midwives acknowledged that the framework’s steps described nothing new in terms of supporting active labor, however it represents a creative way of communication, reminding midwives about the value about what is often taken for granted or forgotten as indicated in the following statement:

*‘Initially, I thought my gosh, it's like, it's nothing new … but actually the LHF is good because it makes what we do official and it's like, there you go, there's a copy and it's on the wall, it gives it value, and it's a constant reminder of what we should be encouraging.’* (Amy)

The LHF promoted awareness among newly qualified midwives and midwifery students of different options for a physiological birth and increased their confidence:

*‘This gives you a bit of confidence … it just gives junior midwives or student midwives a little bit more, like, a role, in early labor, that maybe they wouldn't have had before … without that piece of paper or poster in front of you, you've no definite, this is what I should be doing.’* (Shelley)

Participating midwives considered that LHF through mobilization and squats lunges etc., could help women to get into established labor – naturally and after induction – more quickly, and to better cope with contractions and labor:

*‘We had a primigravida in who did two circuits of the hopscotch. I think it would have taken her, maybe, five hours, and she (her cervix) was fully dilated when she came into the hospital.’* (Brenda)

### Implementation and use of the Labour Hopscotch Framework

Participating midwives reflected on their experiences of using the LHF with women and their birthing partners. They disclosed their preference for the different steps/options presented in the LHF. What became apparent from the discussions was that some of the steps were more widely encouraged and used than others by midwives. Midwives tended to encourage using the birthing ball, stool and regularly mobilizing, while massage and aromatherapy were least used as they were not readily available within the hospital setting. Participating midwives vocalized their awareness of their essential role in helping women to make position adjustments and finding the best-working positions. This showed the flexibility of the LHF:

*‘The ball, the birthing stool and then all fours position ... And then mobilization really worked.’* ( Kim )

Participating midwives highlighted their acknowledgement of the additional choices the LHF gives to many women to take their own initiatives at the prelabor stage before coming to the hospital. As discussions continued, participating midwives disclosed observing several women coming in with the LHF card printed out, either having accessed the information by themselves or being given it during their antenatal appointments:

*‘I think for early labor they do, like antenatally, a lot of them have said they've tried it at home or they're doing different things and that kept them going before they've come in.’* (Shelley)

After the initiation of LHF, participating midwives had witnessed more women using the stairs and doing lunges in the hospital – a phenomenon that was not that common before its implementation:

*‘Yeah, and there's been so many more people out on those stairs recently.’* (Laverne)

*‘I've never seen so many people lunging in (research site) before.’* (Amy)

### Relationships

Participating midwives relayed accounts from their experiences that the LHF was useful in involving birthing partners, which was good for both the women and their partners:

*‘I sent her just a picture of it [Labour Hopscotch] and she was like oh, thanks so much, Richie loves it. The husband. Not even her.’* (Shelley)

Participating midwives indicated that the LHF encouraged partners to touch the laboring woman and to support the woman through the process. This was noted to generate a more positive birth experience for the couples involved:

*‘The fact that dad is so included with the hopscotch, there's less fear and, you know, it's making the woman more relaxed and obviously there's less adrenaline … so it's everything, the big cycle, and how she's feeling emotionally, physically, everything. It's all helping, and I think because the two of them are working together to do it.’* (Samantha)

Participating midwives suggested that women feel less fear and are more relaxed when receiving physical support from their partner when they work together with the LHF. What was very apparent is that midwives considered the LHF supported the birthing partner to take a more active role in the birthing process.

### Barriers and challenges to using Labour Hopscotch

To facilitate the integration of the Labour Hopscotch participating midwives were asked their perspectives on barriers to the use of the LHF. All participants agreed that the main challenge they had experienced was inadequate space and lack of facilities in the hospital setting. The labor ward corridor and back stairs were the only space women could use for walking up and down, when in established labor. There was no access to using birthing balls in the antenatal ward, mainly because there was not enough space. Participating midwives found this to be a particular barrier to utilizing Labour Hopscotch:

*‘Stairs wise, there's no-where else in delivery ward for them, because you can't bring them outside delivery.’* (Carol)

*‘(antenatal ward) just does not have the space for each of the steps because we can only use the corridors just for walking, there is no-where else.’* ( Amy )

Participating midwives also reported facility issues with pools, showers, and toilets. They requested bath-tubs or pools, which at the time of data collection were not available at the research site, and more toilets and showers to be accessible to women:

*‘Facilities I think here can be a problem. Sitting on the toilet for twenty minutes. I know that sounds crazy, but, again, it's not always available. You can't do that in every room.’* ( Samantha )

Despite these barriers, participating midwives explained that they try to make the rooms more birth-friendly by dimming the lights down and using the framework as much as possible with the limited facilities available. Tiredness, however, was deemed to be a further barrier to the use of the framework, as Brenda explained:

*‘But the physicality of it is also sometimes a barrier. And I think that staff tiredness can be a barrier. Or mother tiredness, where a mother says: you can't really ask me to get off this chair again or to sit on that toilet again, or you know, whatever, so that sets a tiredness.’* ( Brenda )

Participating midwives described several personal factors that may influence women’s engagement with LHF, such as fitness levels, birthing philosophy, and expectations. Additionally, primigravida’s were perceived to be more open to Labour Hopscotch than multigravidas by midwives:

*‘I think first-time mothers are more likely to try - Labour Hopscotch … second time mothers especially if the last time they were in, they were induced, epidural, oxytocin. They think that's what labor is, is in the bed and they didn't even know you could be in labor and walk around or use a ball.’* (Shelley)

As the conversation continued, however, it became evident that midwives themselves could be a barrier to promoting LHF to multigravidas, as they could have preconceived assumptions that these women would labor quickly, as highlighted in the conversation below:

*‘As midwives though, we can be barriers for second time mothers getting involved in the hopscotch as well. I think, maybe this isn't all the time, but we know, like, a second-time mother, you're like, she'll probably fly it, so you're less inclined to be, like, do you want to get out of the bed …’* (Samantha)

However, two participants disagreed and placed equal value on the framework for multiparous and primiparous women, especially for the pre-labor stage:

*‘Antenatally, I don't think it makes a difference if they're second or first-time mothers, according to them and I heard a second-time mother saying oh, that's amazing, it's brilliant, and she went through it and it really helped her, but she still wanted her epidural in labor… So, antenatally, it's really good.’* (Amy)

### Education in relation to Labour Hopscotch

To improve student and newly qualified midwives’ confidence in the use of the steps within the Labour Hopscotch, participating midwives recommended introducing LHF training into the midwifery curricula from the start of the education program, and refresher sessions on an annual basis, as indicated in the following statements:

*‘It really does give students a feeling that they can do something, especially in first year when you know nothing.’* (Shelley)

*‘And if you, do it from the start, you're probably more likely to carry it on … Whereas for me to start when I've had, like four years, I'm not doing it as much.’* (Carol)

The conviction in the recommendations about regular refresher classes was evident as all participating midwives discussed the importance of the LHF for midwifery students. This training was deemed imperative for new students to be able to practice and continue its use throughout the years of their studies, as indicated in the following conversation:

*‘See, no point doing it at the end you've seen it, you've worked it. Whereas at the very start when you can't do anything, you'll have a little bit of confidence that you can be like, I can do this with her.’* (Samantha)

*‘Probably no harm in doing it every year, like two hours every year, or a lab, kind of. Even to go through the more technical stuff.’* (Shelley)

Participating midwives also thought it would be beneficial to organize an LHF study day (e.g. four hours) for midwives in the hospital because they might not have received any formal LHF training when they were undertaking their midwifery program.

## DISCUSSION

Midwives often perceive their professional role and responsibility as promoters or protectors of physiology^1^ and seek to, as part of this role, to educate and inform women to enable women to feel empowered to make decisions that are right for them^1,4^. The Labour Hopscotch Framework is an innovative tool that can assist midwives to promote and encourage active labor. Participating midwives in this study welcomed the introduction of the LHF, suggesting it inspired women and their birthing partner to take initiatives and have an active role in their birthing experience. Midwives suggested the LHF was an excellent resource for women in early labor at home and in the hospital setting.

Each step of the LHF (mobilization, birthing stool or toilet, water therapy, birthing ball or mat, alternative therapy) works with physiology to achieve an active labor and in this way support physiology for birth. Facilitating movement and the adoption of different positions has known benefits, namely the effects of gravity^3,5,8^. Alternative therapies are being increasingly used during pregnancy and birth^7^, and as midwives advocate for women and their choices during birth it is important that they are knowledgeable about alternative therapies^10^. The main benefits of choosing alternative (non-pharmacological) pain relief methods are that they are safe, non-invasive, do not produce the side effects that pharmacological methods produce and are easily applicable and inexpensive^17-20^. Massage, for example, can have extremely desirable effects on laboring women, such as a reduction in a woman’s perception of pain, a distraction from pain, reduction in blood pressure and anxiety levels, as well as improvement in maternal mood and feelings of support^21^. Midwives’ use of alternative therapies, such as massage, homeopathy and acupressure, require midwife familiarity with the method, and, in some cases, such as water therapy, facilities and space^21,22^. The current study mirrors the findings describing midwives’ limited knowledge or training in specific alternative therapies and are requesting further training and demonstration for them and women. Furthermore, equipment and resources should be provided to offer these alternative methods to laboring women^22,23^.

Physiological birth in a hospital setting has been described as existing along a board continuum^24^. For example, on occasions when a woman requires intervention, such as induction of labor, midwives can still normalize labor by enhancing the physiological aspects of care^24^. The midwives in the current study described navigating the existing hospital setting, protecting the birth space, and the ease in which most of the LHF steps can be included in a pre-labor or induction process. That said, facilities and space are a barrier to adequately implementing some of the LHF steps in the existing research site, which is severely restricted by space. Furthermore, a shift is required in the way that birthing rooms are furnished and laid out. Midwives, themselves, were suggested by the participants in this study, as possible additional barriers to using the framework, specifically when caring for multiparous women. Previous research has reported similar findings, midwives can exhibit preconceived perceptions and thoughts about alternative birth positions that can either promote or hinder movement in labor. For example, midwives in the study of Musie et al.^25^ stated that they were slow to encourage active labor as the lithotomy position provides a good view of the perineum, is more convenient for fetal monitoring and that it minimizes the midwives physical strain during the birth. These findings and the findings in the current study support the recommendation for further education to improve midwives’ involvement in assisting women with movement during labor to improve the woman’s birthing experience. Education is the key to promoting normal birth. A recent Brazilian study^26^ reported a positive effect of an educational intervention to improve evidence-based practices on normal birth care. Côrtes et al.^26^ noted an increase in the normal birth rate of 5.3% and a reduction in amniotomy, lithotomy position and oxytocin infusion and an increase in partner involvement after their educational intervention. The development of confidence in promoting normal birth is nurtured and cultivated from as early on as midwifery training. As reported in the current study’s findings, Mudokwenyu-Rawdon et al.^27^ reported that educating midwifery students in active labor will enable them to build confidence in providing women with this tangible type of assistance during labor. Midwifery students describe feelings of personal power when the midwifery philosophy of care is supported and expressed in practice^28^.

A key theme throughout midwives’ accounts was the importance of the relationship between the woman and her midwife. Furthermore, an enhancement in the women– partner relationship before and during labor was identified. Such involvement was deemed beneficial, by the midwives, for both mother and partner, because it supported the child birthing process and nourished their relationship. This is important because the international evidence reveals that a supportive birthing partner has also been shown to have a calming effect on the mother and increase her feelings of control during labor and birth^29^.

### Limitations

This study is not without its limitations, as it was undertaken in one maternity unit in Dublin, Ireland. Exploratory research is generally conducted with relatively small sample sizes, such as the current study, but this does not invalidate the findings, gathered from midwives’ perspectives. Women of the research maternity site have access to midwifery-led care, including homebirth, and obstetric-led packages of care, which may not be available in other units. The strength, however, lies in the transferability of the Labour Hopscotch Framework to different home or hospital settings. Additionally, once the LHF is implemented in other units nationally and/or internationally, midwives’ explorations of its implementation in different settings are easily achievable.

## CONCLUSIONS

The Labour Hopscotch is a framework developed to assist midwives in promoting maternal mobility in labor and encouraging optimal fetal positioning, thus enabling midwives to fulfil the philosophy of woman-centered care. The Labour Hopscotch was described as an attractive and creative method of communicating steps which women in labor can take to support choice and steps associated active physiological birth. These findings coupled with the observations from the midwives that the use of Labour Hopscotch increased partner involvement during the labor process is very encouraging. Moving forward, it is therefore an imperative that the wide-spread promotion of Labour Hopscotch is undertaken within maternity care services.

## Data Availability

The data supporting this research are available from the authors on reasonable request.
